# Protective Effects of Two Halophilic Crude Extracts from *Pseudomonas zhaodongensis* and *Bacillus stratosphericus* against Memory Deficits and Anxiety- and Depression-Like Behaviors in Methionine-Induced Schizophrenia in Mice Focusing on Oxidative Stress Status

**DOI:** 10.1155/2020/8852418

**Published:** 2020-11-24

**Authors:** Yousra Massaoudi, Jaouad Anissi, Radu Lefter, Andrei Lobiuc, Khalid Sendide, Alin Ciobica, Mohammed El Hassouni

**Affiliations:** ^1^Biotechnology, Environment, Agri-Food and Health Laboratory, Sidi Mohamed Ben Abdellah University, Faculty of Sciences Dhar El Mahraz, BP: 1796, Atlas, Fez, Morocco; ^2^School of Engineering BIOMEDTECH, Euro-Mediterranean University of Fez, Rond-point Bensouda, Route de Meknès BP 51, Fez, Morocco; ^3^Romanian Academy, Iasi Branch, Center of Biomedical Research, B dul Carol I, 8, 700506 Iasi, Romania; ^4^CERNESIM Research Centre, L2, Alexandru Ioan Cuza University, 700505 Carol I Bd., Iasi, Romania; ^5^Human Health and Development Department, Stefan Cel Mare University, 720229 Universitatii Str., Suceava, Romania; ^6^Laboratory of Biotechnology, School of Science and Engineering, Al Akhawayn University in Ifrane, P.O. Box 104, Ifrane, Morocco; ^7^Department of Research, Alexandru Ioan Cuza University of Iasi, Faculty of Biology, Bd. Carol I, 20A, 700505 Iasi, Romania

## Abstract

Recently, the implication of oxidative stress in behavioral-like disorders has received a lot of attention. Many studies were interested in searching for new natural compounds with protective effects on behavioral-like disorders by focusing on oxidative stress as the main causal factor. Here, we assess the potential effect of cell-free extracts from halophilic bacteria on memory, anxiety, and depression-related behaviors in mice, as well as on cognitive deficits, negative symptoms, and some oxidative stress biomarkers in methionine-induced mice models of schizophrenia. Firstly, crude extracts of bacteria isolated from the Dead Sea were screened for their effects on memory and anxiety- and depression-like behaviors through Y-maze, elevated plus maze, and forced swimming test, respectively, using two doses 60 mg/kg and 120 mg/kg. Then, 120 mg/kg of two bacterial crude extracts, from two strains designated SL_22_ and BM_20_ and identified as *Bacillus stratosphericus* and *Pseudomonas zhaodongensis*, respectively, with significant contents of phenolic and flavonoid-like compounds, were selected for the assessment of cognitive and negative symptom improvement, as well as for their effects on oxidative stress status in methionine-induced mice models of schizophrenia using six groups (controls, methionine, crude extracts solely, and combinations of crude extracts and methionine). Results showed that the administration of the crude extracts caused a significant increase in the spontaneous alternations in the Y-maze task, the time spent in open arms of the elevated plus maze, and a decrease in immobility time in the forced swimming test in comparison with the control group. Furthermore, the administration of bacterial extracts seemed to diminish disorders related to cognitive and negative symptoms of schizophrenia and to improve the oxidative state in the temporal lobes, in comparison with the methionine group. Our findings suggest substantial antioxidant and anti-neuropsychiatric effects of the crude extracts prepared from *Pseudomonas zhaodongensis strain* BM_20_ and *Bacillus stratosphericus strain* SL_22_ and that further studies are needed to purify and to determine the active fraction from the extracts.

## 1. Introduction

During the last decade, a great attention was given to the role of oxidative stress in neuropsychiatric disorders. Many studies have recently demonstrated that the overproduction of free radicals is involved in several behavioral disorders such as short-term memory impairment, anxiety, depression, and schizophrenia [1–4]. In normal aerobic conditions, reactive oxygen species (ROS) are produced as intermediates during metabolic reactions, and then oxidative stress takes place in the presence of altered redox control, resulting in excessive production of ROS usually along with aberrant antioxidant defense mechanisms [5], which might damage the cells by affecting the functional maintenance of major biomolecules (DNA, proteins, and lipids) and gene expression [6, 7]. Moreover, ROS may attack phospholipids and polyunsaturated fatty acids (PUFA) leading to unstable membrane structure and impaired signal transduction and producing severe pathological and toxic species such as malondialdehyde (MDA), a biomarker of lipid peroxidation in living systems [3, 4]. To protect the living systems from ROS damage and toxicity, various enzymatic and nonenzymatic antioxidant pathways are involved to keep their production under tight control [8]. The protective enzymatic pathways act in a cooperative cascade that includes, among others, superoxide dismutase (SOD) and glutathione peroxidase (GPx) [3, 9] in order to reduce the damaging effect of ROS throughout different processes including prevention of ROS formation, scavenging free radicals, preventing the radical chain reaction of oxidation, and/or retarding the lipid peroxidation process [10].

Schizophrenia is a common psychotic disorder characterized by gross distortion from reality [8] and associated with several substantial psychiatric comorbid disorders [11], the most common being depression and anxiety disorders, which contribute to high rate of morbidity and mortality among patients with schizophrenia [12]. Depressive symptoms could be observed in all stages of schizophrenia [13], with a prevalence rate of 55% in the first episode psychosis [14]. On the other hand, anxiety has been considered as a score aspect of schizophrenia, namely, panic and social anxiety symptoms [15]. Prevalence rates are reported between 35 and 65% in patients with schizophrenia [16]. Otherwise, rates of anxiety and depression diagnostic comorbidity vary across the process of schizophrenia [17]. Considering the complex pathophysiology of schizophrenia, different hypotheses have been proposed regarding the etiology, pathophysiology, and routes of treatment of these disorders [18–20]. Currently, evidence sustains that cellular damage of key macromolecules induced by increased oxidative stress levels could play a critical role in schizophrenia. The failure of the antioxidant defense system in protecting against ROS formation damages cell membranes, impacts neurotransmission, and eventually leads to phenotypes of schizophrenia [21]. For deeper studies, symptoms of schizophrenia are generally induced in animal models via overadministration of methionine for 7 and 15 days [22]. Hence, it was reported that methionine is able to replicate both positive and negative symptoms, as well as cognitive deficits of schizophrenia [19, 22, 23]. It is worth recalling that methionine (Met) is an essential amino acid required for S-adenosyl methionine (SAM) generation and in almost all methylation reactions of a very wide range of substrates including proteins, phospholipids, RNA, and DNA [24]. In turn, SAM is a major methyl donor that influences the central nervous system (CNS) function through several methylation reactions, the most relevant being deactivation via methylation of multiple neurotransmitters, epigenesis through methylation of key molecules involved in gene expression (DNA, RNA, and histones), and methylation of phospholipids [25]. In addition, methionine is considered as an intermediate substrate for the synthesis of some amino acids such as homocysteine, a nonessential amino acid that is involved in oxidative stress and cognitive dysfunctions [26], formed via demethylation of L-methionine [27]. Recent studies have shown that chronic administration of methionine induces hyperhomocysteinemia and contributes to subsequent cognitive function deterioration, which could be attributed to increased levels of oxidative stress and lipid peroxidation by hyperhomocysteinemia [28, 29]. It is well known that cellular hyperhomocysteinemia causes autooxidation of thiol groups that generate hydrogen peroxide and other ROS leading to oxidative stress [30]. Overall, Met is considered as a main factor in the etiology of schizophrenia [22]. Treatment of schizophrenic symptoms is mainly based on approved antipsychotic drugs. However, long-term use of antipsychotics has limited efficacy and has been associated with various side effects such as weight gain, metabolic disturbances, oxidative stress, sudden cardiac death, and cognitive impairment, which highly limit their clinical use [20]. Therefore, developing novel antipsychotic drugs with better safety and efficacy is crucial. In this field, some studies have focused on seeking new natural compounds from medicinal plants with promising value in the treatment of neuropsychiatric disorders [31–33], but studies assessing bacterial metabolites for their therapeutic effects as neuropsychiatric behaviors modulating agents remain relatively limited [34–36].

Halophilic bacteria are defined as microorganisms with high ability to live in very saline biotopes [37] and have been widely reported for their capacity of producing promising biocompounds with a large spectrum of activities [38, 39] including antioxidant, anticancer, and immunomodulatory activities [39–41]. Nonetheless, to our knowledge, no data have been reported on the effects of halophilic bacteria on schizophrenic-like symptoms. Considering the pharmaceutical importance of metabolites produced by halophilic bacteria in harsh conditions and their powerful antioxidant activity and in view of the little data reported on the effects of these halophilic bacterial extracts on neuropsychiatric disorders [42], the present study aims at isolating halophilic bacteria producing metabolites that could potentially act on short-term memory impairment, anxiety, depression-like behaviors, and schizophrenia-like disorders in animal models. Some oxidative stress biomarkers were also considered.

## 2. Materials and Methods

### 2.1. Bacteria Isolation

Littoral soil and water samples were collected from Dead Sea (Jordan) at the following global positioning system coordinates: 31°41′18.29″N; 35°34′55.72″E. Bacteria were isolated from the soil by suspending 1 g of soil in 100 ml of sterile Luria Bertani medium (LB). Serial dilutions from the suspension were platted on LB agar medium containing concentrations of sodium chloride (NaCl) ranging from 0 to 3 M. Growth was performed at 30°C for 72 hours, and strains were selected and stored for further use.

### 2.2. Strain Characterization and Identification

Strains were characterized by their phenotypic and biochemical characteristics according to the standard methods described in Bergey's Manual of Systematic Bacteriology [43]. The growth at different NaCl concentrations ranging from 0 to 3 M was performed in LB medium, and the growth was maintained separately at 30°C and 37°C. Based on rRNA gene amplification and sequencing, the 16S rRNA gene was amplified using the recommended universal primers, 27F (AGAGTTTGATCCTGGCTCAG) and 1392R (GGTTACCTTGTTACGACTT), and amplification was conducted as described by Turner et al. [44]. Sanger sequencing was performed at the Center of Innovation (USMBA, Fez-Morocco) using an ABI PRISM 3130XL genetic analyzer (Applied Biosystems). Sequences were compared with 16S rRNA gene sequences available in the GenBank database. Phylogenetic relationships were established using the neighbor-joining (NJ) criteria under MEGA X software [45]. Phylogeny tests were assessed by bootstrapping with 1000 replicates, and the maximum likelihood composite was used as a substitution model.

### 2.3. Bacterial Culture and Crude Extract Preparation

A preculture of each strain was grown to an OD_600nm_ 0.8–1 in GYM medium (glucose 4 g/l, yeast extract 4 g/l, and malt extract 10 g/l), and 1 ml of the preculture was then used to inoculate 100 ml of GYM medium. After 48 h–72 h of incubation at 30°C under agitation (at 150 rpm), the fermentation broth was centrifuged at 9000*g* for 10 min, and the supernatant was filtered through a 0.22 *μ*m nitrocellulose filter. The filtered supernatant was then brought to pH 3.0 and loaded onto a preequilibrated nonionic resin type Amberlite XAD-4-containing column. The adsorbed material was eluted with methanol as a mobile phase. Fractions were collected and dried at 40°C using a rotary evaporator. The crude dried extracts were weighed and then prepared at a concentration of 500 mg/ml in sterile distilled water for further analysis.

### 2.4. Determination of Phenolic Contents and Antioxidant Activities of the Crude Extracts

Phenolic-containing and flavonoid-like compounds present in the crude extracts were determined using the methods described by Singleton et al. and Dewanto et al. [46, 47], and the obtained results were expressed as *μ*g of acid gallic equivalent and *μ*g of quercetin equivalent per mg of crude extract, respectively. The antioxidant activities of the crude extracts were assayed using 2,2-diphenyl-1-picrylhydrazyl (DPPH) scavenging activity and molybdenum Mo(VI) reducing power [48, 49]. The obtained results were expressed as *μ*g of ascorbic acid equivalent per mg of the crude extract. The percentage of DPPH scavenging activity was calculated according to the following formula:

(1) $$\begin{array}{c} \text{DPPH scavenging activity}\left(\%\right)=\frac{\left(A_{0}-A_{1}\right)}{A_{0}}\times 100, \end{array}$$

where *A*_0_ represents the absorbance of control (DPPH solution) and *A*_1_ represents the absorbance of the experimental, respectively.

### 2.5. Animals

In all experiments, male adult Swiss mice weighing 25 g to 30 g at the beginning of the experiments and randomly distributed throughout the study groups described below were used. Animals were maintained in constant environmental conditions (a temperature of 22 ± 1°C, 55–60% humidity, and natural light-dark cycle, with food and water ad libitum) in polyacrylic cages (6 animals/cage) containing woodchip bedding. The habitation behavior was assessed 15 days before the experiments, observing appetite, water intake, digestive transit, and neurologic signs and behavior (e.g., socialization and group behavior). Laws on animal use in biomedical research were considered in animal care and experimental procedures.

### 2.6. Behavioral Studies

#### 2.6.1. General Experimental Design

**Figure 1:**
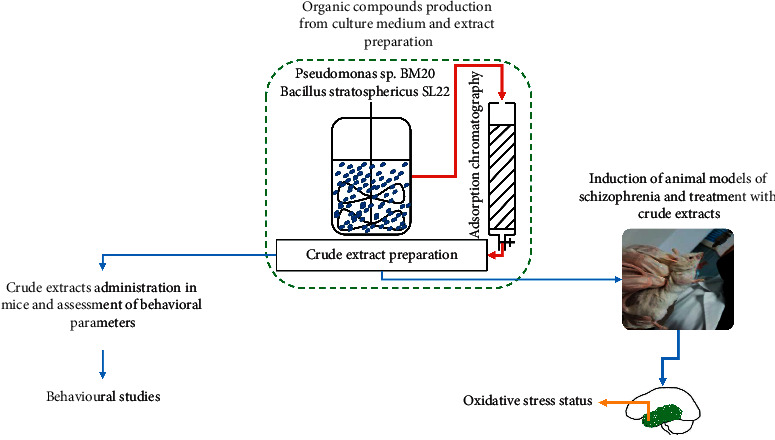
Schematic pattern explaining the general design of the study. (1) Preliminary screening of crude extracts for their effects on modulating short-term memory and anxiety- and depression-like disorders. (2) Induction of schizophrenia by overadministration of methionine and assessment of effects of crude extracts on both cognitive deficits and negative symptoms of schizophrenia-like disorder. (3) Assessment of crude extracts' effect on the oxidative stress status in the temporal lobe of methionine-induced mice models of schizophrenia.

The present study consists of two major parts. In the first part, crude extracts were tested for their effects on modulating short-term memory and anxiety- and depression-like behaviors using Y-maze, elevated plus maze, and forced swimming test, respectively (Figure 1). To do so, two doses of the crude extracts were prepared (60/kg and 120 mg/kg) in sterile distilled water and administered orally to a group of 30 mice divided into 5 groups of 6 mice (Table 1). Animals (*n* = 30) were fed and allowed to drink water at a specific time (7:00 p.m.) each day during the entire administration period (7 days). Each mouse was orally administered 80 *μ*l of the corresponding tested crude extract (SL_22_ and BM_20_) once a day for 7 days. The “control” group was kept in the exact same conditions and administered by the same volume of physiological water. Behavioral experiments began the following day after treatment. All behavioral assessments were carried out between 9:00 a.m. and 3:00 p.m.

In the second part of the experiment, we aimed at investigating the effect of crude extracts on schizophrenic symptoms in a methionine-generated schizophrenia mouse model by using the previous bacterial extracts of the highest concentration (120 mg/kg). To this end, a set of 31 mice was used, which were distributed randomly in six groups (*n* = 5 to 6 mice per group), kept in a room with controlled temperature (22 ± 1°C) with food and water ad libitum (Table 2). The animal model of schizophrenia was induced with methionine via daily subcutaneous injections of 50 *μ*l of methionine (37.5 mg/ml) for 7 days. At the end of the methionine treatment, 50 *μ*l of each extract was orally administrated to the corresponding group for 5 consecutive days. Short-term memory, anxiety, and depression-like manifestations were evaluated using the Y-maze and novel object recognition test, elevated plus maze, and forced swimming test, respectively (Figure 1).

#### 2.6.2. Short-Term Memory and Cognitive Deficit Evaluation

*(1) Y-Maze*. The short-term memory performance was evaluated by recording the spontaneous alternation behavior in the Y-maze. The maze consists of three opaque arms (15 cm long, 3 cm wide, and 5 cm high), in addition to an equilateral central area. Each mouse was placed at the end of one arm and allowed to move freely through the maze for 8 min, during which the spontaneous alternations between the arms were recorded and the spontaneous alternation percentage was calculated using the following formula:

(2) $$\begin{array}{c} \text{spontaneous alternations}=\left(\frac{\text{number of correct alternations}}{\text{number of total alternations}-\;2}\right)\times 100. \end{array}$$

A spontaneous alternation is defined as the entry in three consecutive different arms. An arm entry is counted when the mouse has entered within the arm with all four paws. The maze was cleaned with alcohol-free disinfectant wipes between each trial [50].

*(2) Novel Object Recognition Test*. NOR is a highly validated test for the evaluation of cognition, particularly the recognition memory in rodent models of central nervous system disorders. The apparatus used for this test is an open wooden box 40 cm long, 40 cm wide, and 40 cm high. The novel object recognition test is a nonforced memory test, in which any environmental stressor or stimulation can increase the results' variability. In this regard, a two-day acclimatization procedure was conducted during which mice were first transported in the operative chamber and allowed to habituate for 10 min. Then, each mouse was put in the apparatus individually and let to analyze, habituate, and acclimate with the environment for 10 min. On the third training day, the same procedure was repeated, but with two identical objects (4 cm*∗*4 cm*∗*4 cm) placed in the box which the mice were allowed to explore freely for 10 min. On the fourth day, which was the testing day, one of the familiar objects was randomly replaced by a novel different object, and each mouse was allowed to explore again the box for 10 min. The test is based on the spontaneous tendency of mice to spend more time exploring the novel object than the familiar one [51]. The percentage of preference and discrimination index (%) was calculated using the following formulas:

(3) $$\begin{array}{c} \text{percentage of preference}=\left(\frac{\text{time spent to explore the novel object}}{\text{total time spent to explore both objects}}\right)\times 100,\\ \text{discrimination index}\left(\%\right)=\left(\frac{\text{time}\left(\text{novel}\right)-\text{time}\left(\text{familiar}\right)}{\text{time}\left(\text{novel}\right)+\text{time}\left(\text{familiar}\right)}\right)\times 100. \end{array}$$

#### 2.6.3. Anxiety Behavior Assessment

The elevated plus maze (EPM) was used to assess anxiety. The maze consists of four arms (26 cm long and 2 cm wide), elevated at 15.25 cm above the ground. Two arms are opened and two closed by walls with a juncture in the middle. Each mouse was placed in the juncture and allowed to explore freely the maze for 5 min. The time spent in the open arms is an index of anxiety [52]. Increased entries in the open arms reflect exploratory behavior and reduced anxious behavior.

#### 2.6.4. Depression Behavior Assessment

The forced swimming test (FST) was used to evaluate the antidepressant effects of the bacterial extracts. Mice were placed individually into a cylindric recipient (30 cm wide and 59 cm high) containing 30 cm of lukewarm water. An adapted protocol version of the test was used, consisting of a 6 min swimming session for each mouse, first two minutes for acclimatization and the last four minutes for measuring. Two factors were evaluated: swimming time and floating time (including minimal movement to keep the head above the water). Floating time was considered as an indicator of depressive state [50]:

(4) $$\begin{array}{c} \text{immobility time}\left(\text{s}\right)=\left(240\text{ seconds }–\text{ swimming time }\left(\text{s}\right)\right). \end{array}$$

### 2.7. Tissue Collection and Antioxidant Enzyme Activity Estimation

After the behavioral tests, all mice were anesthetized, quickly decapitated, and the whole brain was removed (Figure 1). The temporal lobes, considered as the most vulnerable cortical area of the brain to the modifications of the oxidative stress status [8], were collected. Each temporal tissue sample was weighed and homogenized with a Potter Homogenizer coupled with Cole-Parmer Servodyne Mixer in bidistilled water (1 g tissue per 10 ml of bidistilled water). Samples were centrifuged at 1000*g* for 15 min. Then, the supernatant was separated and pipetted into tubes [53].

#### 2.7.1. Determination of Superoxide Dismutase

Superoxide dismutase (SOD) activity was measured by the percentage of reaction inhibition rate of the enzyme with WST-1 substrate (a water soluble tetrazolium dye) and xanthine oxidase using an SOD assay kit (Fluka, product number: 19160) according to the manufacturer's instructions. In the reaction, xanthine oxidase (XO) catalyzes the oxidation conversion of xanthine to yield superoxide anion, which quenches WST-1 to produce water-soluble formazan (a purple dye). Once SOD quenches superoxide anion, its activity inhibits the overall colorimetric reaction, which reflects the SOD activities in the tested tissues. Each endpoint assay was monitored by absorbance at 450 nm (the absorbance wavelength for the colored product of WST-1 reaction with superoxide) after 20 min of reaction time at 37°C. The percent inhibition was normalized by mg protein and presented as SOD activity units [54].

#### 2.7.2. Determination of Glutathione Peroxidase

Glutathione peroxidase (GPx) catalyzes the reduction of hydroperoxides including hydrogen peroxide (H_2_O_2_), by reduced glutathione, and functions to protect cells from oxidative damage. GPx activity was measured indirectly using the GPx cellular activity assay kit CGP-1 (Sigma Chemicals). This kit uses an indirect method based on the oxidation of glutathione (GSH) to oxidized glutathione (GSSG) catalyzed by GPx, which is then coupled with recycling GSSG back to GSH utilizing glutathione reductase (GR) with NADPH consummation. The decrease in NADPH at 340 nm during oxidation of NADPH to NADP^+^ is proportional to the GPx activity [54].

#### 2.7.3. Determination of Malondialdehyde

In order to assess the effect of the tested bacterial extracts on lipid peroxidation status in the collected temporal lobe tissues, malondialdehyde (MDA) biomarker levels were estimated. MDA levels were determined by the thiobarbituric acid reactive substances (TBARs) assay. 200 *μ*l of supernatant were added and briefly mixed with 1 ml of trichloroacetic acid at 50%, 0.9 ml of Tris-HCl (pH 7.4), and 1 ml of thiobarbituric acid 0.73%. After vortex mixing, samples were maintained at 100°C for 20 min. Afterwards, samples were centrifuged at 1000*g* for 10 min and supernatant read at 532 nm. The signal was read against an MDA standard curve, and the results were expressed as nM/mg protein [55].

### 2.8. Ethical Approval

All procedures performed in studies involving animals were in accordance with the guidelines of animal's bioethics from the Act on Animal Experimentation and Animal Health and Welfare Act from Romania, and all procedures were in compliance with the European Council Directive of 24 November 1986 (86/609/EEC) and were approved by the Local Committees with the registration number 3473/2017.

### 2.9. Data Analysis

Data were analyzed using one-way analysis in variance (ANOVA) in Excel and presented as “mean ± SD.” Groups were compared against each other and against the control group. *F* values were considered of statistical significance at a value of *P* < 0.05.

## 3. Results

### 3.1. Strain Isolation and Identification

The preliminary screening of strains from the Dead Sea for their ability to produce secondary metabolites with potential therapeutic outcome in neuropsychiatric behaviors allowed us to select two halophilic strains, designated SL_22_ and BM_20_. The strains were characterized based on their morphological, physiological, and biochemical characteristics. Biochemical characteristics of the two isolates are presented in Table 3 in comparison with the closest species. Phylogenetic analysis based on 16S rRNA sequences clustered the strain SL_22_ within the genus *Bacillus* among the group of *B. stratosphericus*, *B. licheniformis, B. sonorensis, B. aerius, and B. aerophilus* [56] and the strain BM_20_ among the genus *Pseudomonas* within the group of *P. zhaodongensis*, *P. xanthomarina, P. kunmingensis, and P. stutzeri* [57]. 16S rRNA sequences of SL_22_ and BM_20_ strains are deposited under the GenBank accession numbers MN197843 and MK503774, respectively. Strain BM_20_ was deposited in the DSMZ collection under the number DSM 109191.

### 3.2. Phenolic Contents and Antioxidant Activities of the Bacterial Crude Extracts

The extracts prepared from the culture medium of the isolated strains were analyzed for their phenolic and flavonoid contents, and results are summarized in Table 4. The extracts from the strains grown in GYM showed phenolic compound contents ranging from 5.35 ± 0.01 *μ*g/mg to 30.80 ± 0.16 *μ*g/mg of gallic acid equivalent. Content in flavonoid-like compounds showed values ranging from 2.50 ± 0 *μ*g/mg to 12.40 ± 0 *μ*g/mg of quercetin equivalent. Furthermore, the antioxidation potential of the extracts was assessed for their scavenging free radical DPPH (2,2-diphenyl-1-picrylhydrazyl) assay and their ability to reduce Mo(VI) to Mo(V) in the phosphomolybdenum assay. From the results displayed in Table 4, BM_20_ showed the highest percentage of scavenging activity (81.84 ± 0.06)%. Among all the tested bacterial extracts, the highest Mo (VI) reducing activity was shown by two crude extracts, which are BM_20_ (18.2 ± 0.06) *μ*g/mg and SL_22_ (17.9 ± 0.13) *μ*g/mg of ascorbic acid equivalent. Based on the obtained results, namely, total phenolic and flavonoid contents, two crude extracts (SL_22_ and BM_20_) were chosen to further continue the study.

### 3.3. Behavioral Parameters

#### 3.3.1. Effect of the Bacterial Crude Extracts on Short-Term Memory

**Figure 2:**
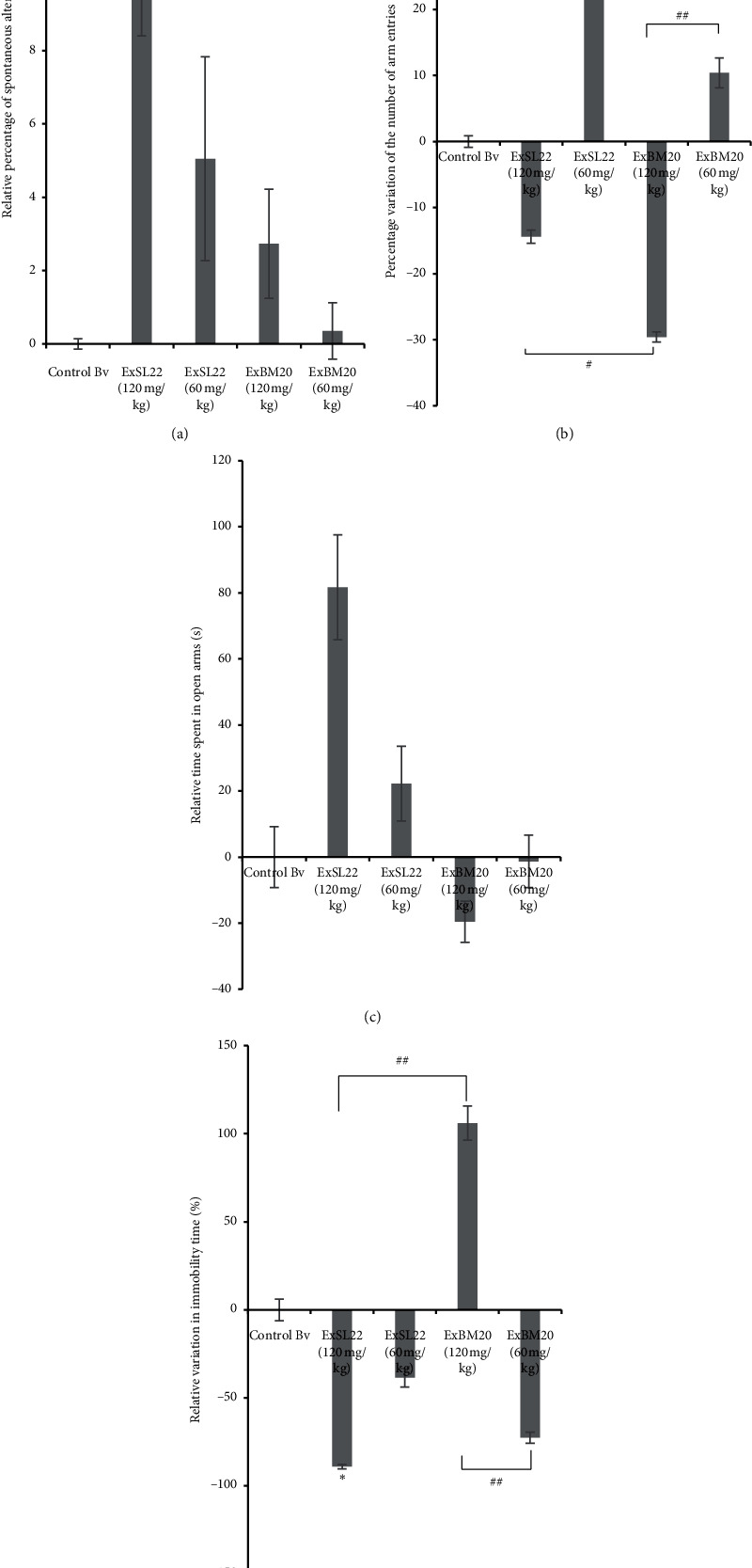
Effect of the bacterial crude extracts on the behavioral parameters, namely, on short-term memory enhancement, on anxiety, and on depression-like behaviors. Histogram shows variations in relative behaviors as function of the administered extracts. (a) Percentage variation of the relative activity of spontaneous alternations in the Y-maze test under the experimental conditions (extract SL22 120 mg/kg vs. extract BM20 60 mg/kg) (*P*=0.032). (b) Percentage variation in the number of arm entries in the Y-maze test (extract SL22 120 mg/kg vs. extract SL22 60 mg/kg) (*P*=0.006), (extract SL22 120 mg/kg vs. extract BM20 120 mg/kg) (*P*=0.028), (extract SL22 60 mg/kg vs. extract BM20 120/mg kg) (*P*=0.007), and (extract BM20 120 mg/kg vs. extract BM20 60 mg/kg) (*P*=0.005). (c) Percentage variation of the relative activity of time spent in open arms in the elevated plus maze test under the experimental conditions. (d) Relative variation in immobility time (%) under the experimental conditions (extract SL22 120 mg/kg vs. controls) (*P*=0.028), (extract SL22 120 mg/kg vs. extract BM20 120 mg/kg) (*P*=0.004), (extract SL22 60 mg/kg vs. extract BM20 120 mg/kg) (*P*=0.037), and (extract BM20 120 mg/kg vs. extract BM20 60 mg/kg) (*P*=0.009) (^*∗*^*P* < 0.05 vs. controls; ^#^*P* < 0.05 and ^##^*P* < 0.01 treated groups vs. each other) (*N* = 6 animals per group).

As displayed in Figure 2(a), the results from the Y-maze test showed that both crude extracts, SL_22_ and BM_20_, were able to increase the relative variation of the spontaneous alternation percentage in a dose-dependent manner. Thus, we observed an important increase of the spontaneous alternation parameter in the case of SL_22_ extract with a variation of 11.01 ± 2.61% vs. control for the dose of 120 mg/kg. BM_20_ extract showed a relatively lower effect with a variation of 2.73 ± 1.49% vs. control at 120 mg/kg, but it still led to increasing memory performance when compared to the control group. Furthermore, it is interesting to mention that the low dose of 60 mg/kg induced a relative increase in the number of arm entries relative to control for both SL_22_ and BM_20_ extracts (Figure 2(b)). The higher dose of 120 mg/kg led to a remarkable decrease in the number of arm entries in both extract groups (SL_22_ 120 mg/kg vs. SL_22_ extract 60 mg/kg) (*P*=0.006) and (BM_20_ 120 mg/kg vs. BM_20_ 60 mg/kg) (*P*=0.005).

#### 3.3.2. Effect of the Bacterial Crude Extracts on Anxiety-Like Behavior

The assessment of anxiety-like behaviors in the elevated plus maze test (EPM) showed that mice administered with extract SL_22_ at 60 mg/kg and 120 mg/kg spent more time in the open arms in comparison with the control group with values of the relative time spent in the open arms of 31.17 ± 11.34 (s) and 46.33 ± 15.86 (s), respectively vs. 25.5 ± 9.23 (s) control time (*P* > 0.05) (Figure 2(c)). Mice treated with BM_20_ extract showed a decrease in the time spent in open arms compared to the control group, but differences were not statistically significant (*P* > 0.05). Interestingly, animals administered with the higher dose (120 mg/kg) of BM_20_ extract spent less time in the open arms compared to the group administered with 60 mg/kg of extract BM_20_ and compared to the control group, suggesting a possible anxiogenic effect of the BM_20_ extract at high concentration. Therefore, based on our results, we suggest a possible anxiolytic effect of the SL_22_ extract, which seemed able to diminish anxiety-like behavior. Moreover, both crude extracts showed a dose response effect.

#### 3.3.3. Effects of the Bacterial Crude Extracts on Depression-Like Behavior

As shown in Figure 2(d), the extract SL_22_ significantly reduced the immobility time of mice at 60 mg/kg and 120 mg/kg compared to the control group with a probability of *P*=0.028, suggesting an antidepressant effect of this extract. Interestingly, when administered at 120 mg/kg, the BM_20_ extract induced an increase in the immobility state of the animals relative to control, while the same extract at 60 mg/kg induced a passivity state of the mice during the task versus controls (*P* > 0.05). The results suggest beneficial antidepressant activity of SL_22_ extract on the one hand and the possibility that BM_20_ extract could prevent depressive manifestations in moderate doses on the other hand, even if higher doses of the latter may cause depression.

### 3.4. Effect of Bacterial Crude Extracts on Methionine-Induced Mice Models of Schizophrenia

#### 3.4.1. Effects of the Bacterial Crude Extracts on Modulating Memory Deficits in Methionine-Induced Models of Schizophrenia

**Figure 3:**
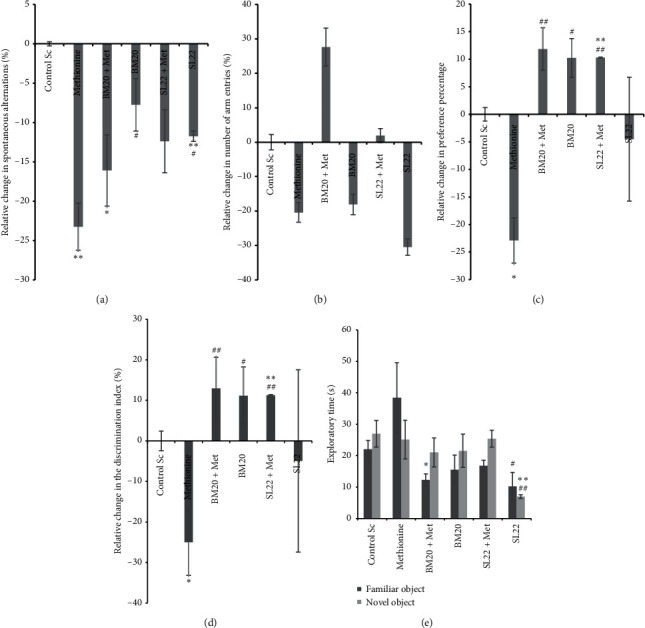
Effect of the bacterial crude extracts on cognitive deficits in methionine-induced mice models of schizophrenia. Histograms show the following parameters. (a) Relative variation change in spontaneous alternation percentage in the Y-maze test under experimental conditions (BM20 + Met vs. controls) (*P*=0.041), (BM20 vs. methionine) (*P*=0.026), and (SL22 vs. methionine) (*P*=0.036). (b) Relative variation in the percentage of the number of arm entries in Y-maze under experimental conditions. (c) Relative variation in the percentage of preference tendency to the novel object was taken in the NOR test under experimental conditions. (d) Relative change in the discrimination index (%) under experimental conditions (BM20 vs. methionine) (*P*=0.010) and (BM20 + Met vs. methionine) (*P*=0.009). (e) Time spent by mice in exploring both familiar (SL22 vs. methionine) (*P*=0.046) and (BM20 + Met vs. controls) (*P*=0.02) and novel objects (SL22 vs. SL22 + Met) (*P*=0.0001), (SL22 vs. BM20) (*P*=0.025), (SL22 vs. BM20 + Met) (*P*=0.016), (SL22 vs. methionine) (*P*=0.018), and (SL22 vs. controls) (*P*=0.001) as assessed in the NOR test (^*∗*^*P* < 0.05,  ^*∗∗*^*P* < 0.01 vs. controls; ^#^*P* < 0.05,  ^##^*P* < 0.01 vs. methionine) (*N* = 5 to 6 per group).

Data related to the immediate spatial memory assessed in the Y-maze task showed a decrease in the relative spontaneous alternation percentage in methionine-treated animals (*P*=0.001). Groups administered with SL_22_ extract (120 mg/kg) + methionine (62.5 mg/kg) did not show any improvement of the immediate spatial memory. In contrast, administration of the same crude extract solely induced a reduction in the spontaneous alternation percentage by 11.74 ± 0.67% vs. control (*P*=0.005). Groups administered with BM_20_ extract at 120 mg/kg exhibited an improvement of the immediate spatial memory compared to the control groups and to the groups administered with the combination (BM_20_ extract + methionine) (*P* > 0.05) (Figure 3(a)). Compared to the group treated with methionine, treatment with crude extracts from isolates SL_22_ and BM_20_ showed a significant increase in the variation of spontaneous alternation percentage. Moreover, Met + SL_22_ and Met + BM_20_ groups were more active and recorded a high number of arm entries in Y-maze compared to methionine and control groups (*P* > 0.05) (Figure 3(b)). These results speculate the possible effect of the extracts on improving short-term memory. Nonetheless, the groups administered with only BM_20_ or SL_22_ did not show any difference compared to the methionine group. Based on these results, we could suggest a possible improved effect of the tested crude extracts when combined with methionine, which could be attributed, at least in part, to the physiological and/or metabolic mechanisms of action of these extracts.

The assessment of cognitive memory in the NOR test revealed that the injection of methionine resulted in a significant decrease of both preference percentage and discrimination index regarding the exploration tendency of the novel object by the treated mice 22.90 ± 4.11% and 24.98 ± 8.21%, respectively, vs. controls (*P*=0.019). However, the treatment with both BM_20_ extract solely and methionine + BM_20_ seemed to increase the preference percentage as compared to both methionine-treated and control groups (10.23 ± 3.54)% (*P*=0.010 vs. methionine) and (11.86 ± 3.86)% (*P*=0.009 vs. methionine), respectively (Figure 3(c)). Furthermore, the two extracts increased the discrimination tendency of the novel object by the corresponding treated mice as compared to both methionine (*P* < 0.05) and control (*P* > 0.05) groups (Figure 3(d)). The injection of SL_22_ solely did not show any significant improvement in comparison with controls. However, when administering SL_22_ + methionine, both preference and discrimination percentages increased significantly compared to both control (*P*=0.001) and methionine (*P*=0.002) groups (Figure 3). As shown in Figure 3(e), the injection of methionine resulted in higher exploratory tendency as compared to controls and mice treated with crude extracts, which could be attributed to a hyperlocomotion state in line with the psychotic effect of methionine. Moreover, as compared to methionine, both controls and treated groups showed a higher tendency to explore the novel object. The results suggest that both bacterial crude extracts improve cognition (cognitive) memory in the treated mice.

#### 3.4.2. Effects of the Bacterial Crude Extracts on Anxiety in Methionine-Induced Mice Models of Schizophrenia

**Figure 4:**
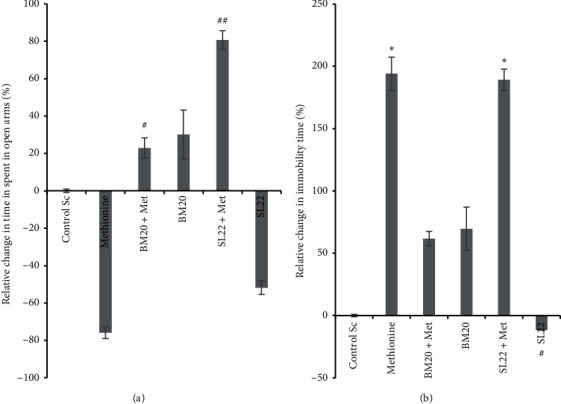
Effect of the bacterial crude extracts on negative symptoms in methionine-induced mice models of schizophrenia. Histograms show the following parameters. (a) Behaviors in the elevated plus maze test were taken as response. The percentages were calculated as the relative percentage to the control value (BM20 + Met vs. methionine) (*P*=0.030) and (SL22 vs. SL22 + Met) (*P*=0.006). (b) Relative change in immobility time (%) as assessed in the forced swimming task (BM20 + Met vs. SL22 + Met) (*P*=0.035) and (BM20 + Met vs. SL22) (*P*=0.032) (^*∗*^*P* < 0.05 vs. controls; ^#^*P* < 0.05,  ^##^*P* < 0.01 vs. methionine) (*N* = 5 to 6 per group).

The results obtained in the elevated plus maze showed that mice administered with methionine revealed a nonsignificant decrease of time spent in the open arms compared to the control group (*P* > 0.05). Besides, as compared to control and methionine groups, mice administered with methionine plus BM_20_ or SL_22_ extracts (*P* < 0.05 vs. methionine) and with extracts only showed an increase in the time spent in the open arms except for the SL_22_ group (*P* > 0.05). The highest significant period spent in the open arms was the one recorded by mice administered with methionine + SL_22_ (30 ± 4.92) seconds (*P*=0.002 vs. methionine) (Figure 4(a)). Nevertheless, the increased open arms time showed by either SL_22_ + Met, BM_20_, or BM_20_ + Met suggest a possible anxiolytic-like effect of the bacterial crude extracts.

#### 3.4.3. Effects of the Bacterial Crude Extracts on Depression in Methionine-Induced Mice Models of Schizophrenia

As mentioned in Figure 4(b) and compared to controls, methionine administration resulted in a significant increase of immobility time (*P*=0.032). This is observed also for the other groups except for the SL_22_ extract solely group (*P*=0.012 vs. methionine), in which mice were able to swim during most of the test time, even more than the controls. Comparing the results of the bacterial extract groups, we observed that BM_20_ and BM_20_ + Met groups did not show a significant difference even if the administration of BM_20_ + Met seemed to reduce the immobility time. The results suggest a possible antidepressant effect of BM_20_ extract when combined with methionine. Nonetheless, compared to controls, BM_20_ seemed to have a depressant-like effect on mice while SL_22_ appeared to have the opposite effect on mice since the group treated with SL_22_ + Met was floating for most of the test time (*P*=0.010 vs. controls), and the results obtained were nearly similar to those of the Met group (Figure 4(b)). However, mice receiving SL_22_ extract solely swam longer than the group treated with SL_22_ + Met (*P*=0.001) and the control group (*P* > 0.05). Therefore, we suggest a possible antidepressant effect of the SL_22_ bacterial crude extract in methionine-induced mice models of schizophrenia.

### 3.5. Enzymatic Antioxidant Activity Estimations

**Figure 5:**
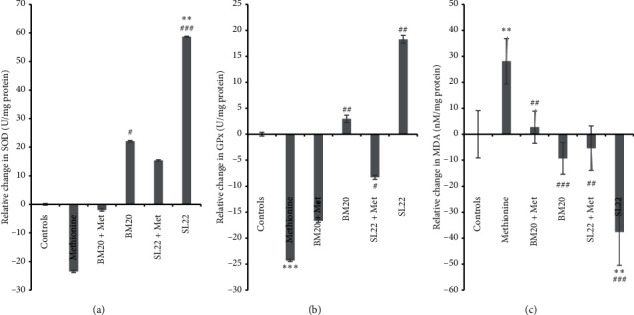
Effects of methionine-induced mice model of schizophrenia and halophilic bacterial crude extract administration on the enzymatic antioxidant activity estimations. (a) Relative change in the superoxide dismutase (SOD) specific activity in the temporal lobe (BM20 + Met vs. SL22) (*P*=0.008) and (BM20 vs. SL22) (*P*=0.025). (b) Relative change in the glutathione peroxidase (GPx) specific activity in the temporal lobe (BM20 + Met vs. controls) (*P*=0.011), (BM20 vs. methionine) (*P*=0.008), (BM20 + Met vs. BM20) (*P*=0.045), (BM20 + Met vs. SL22) (*P*=0.004), and (SL22 + Met vs. SL22) (*P*=0.021). (c) Relative change in the malondialdehyde (MDA) concentration in the temporal lobe (BM20 + Met vs. methionine) (*P*=0.006), (SL22 + Met vs. methionine) (*P*=0.002), (BM20 + Met vs. SL22) (*P*=0.002), and (BM20 vs. SL22) (*P*=0.014) (^*∗*^*P* < 0.05,  ^*∗∗*^*P* < 0.01,  ^*∗∗∗*^*P* < 0.001 vs. controls; ^#^*P* < 0.05,  ^##^*P* < 0.01,  ^###^*P* < 0.001 vs. methionine) (*N* = 5 per group).

Regarding the oxidative stress markers, when we analyzed the effect of the tested crude extracts on SOD specific activity in the temporal lobe's samples, we noticed a decrease in SOD activity by 23.47 ± 0.38 U/mg protein in the Met group versus controls (*P* > 0.05). A significant increase of SOD activity was observed in the SL_22_ group (58.67 ± 0.21 U/mg protein) compared to the controls (*P*=0.001 vs. controls and *P*=0.0008 vs. methionine group) and to the SL_22_ + methionine group (15.37 ± 0.27 U/mg protein) (*P*=0.008), which suggests an effect of SL_22_ extract on SOD activity in the brain of the treated mice. Conversely, BM_20_ seemed to increase the SOD activity in the brain of the treated mice (22.08 ± 0.30 U/mg protein) in comparison with control and methionine (*P*=0.032) groups and with the BM_20_ + methionine group (1.93 ± 0.43 U/mg protein) (*P* > 0.05) (Figure 5(a) and Table 5). These results highlight the positive effect of the halophilic crude extracts on SOD specific activity, which acts as one of the primary antioxidant enzymes by converting superoxide to hydrogen peroxide, which is then decomposed to water and oxygen by catalase (CAT).

As shown in Figure 5(b), the injection of methionine resulted in a significant reduction of GPx specific activity of the treated animals by −24.30 ± 0.19 U/mg protein compared to the control and with a probability of *P*=0.0006, while administration of SL_22_ extract led to an increase in the GPx specific activity in the corresponding animals by 18.27 ± 0.76 U/mg protein, compared to controls (*P* > 0.05) and to the methionine group (*P*=0.001). Mice treated with a combination of SL_22_ + methionine manifested a decrease in the GPx specific activity as measured in the temporal lobe (8.28 ± 0.41 U/mg protein) (*P* > 0.05 vs. control and *P*=0.010 vs. methionine groups). We further noticed that BM_20_ exhibited a significant effect on GPx activity in the treated animals (2.96 ± 0.71 U/mg protein) as compared to control and methionine (*P* < 0.05) groups or to the BM_20_ + methionine group (16.65 ± 0.29 U/mg protein) (*P* < 0.05).

We further examined the levels of lipid peroxidation products by measuring MDA levels, the end product of lipid peroxidation, in the temporal lobe of treated mice. Obtained results (Figure 5(c)) showed a significant increase of MDA levels in methionine-treated mice by 28.12 ± 8.76 nM/mg protein compared to the controls (*P*=0.008), which points to a possible cause of lipid peroxidation by methionine administration in the corresponding mice. On the other hand, BM_20_ administration reduced MDA levels in the corresponding animals (9.29 ± 6.11 nM/mg protein) as compared to control (*P* > 0.05) and methionine groups (*P*=0.0006) or to the BM_20_ + Met group (2.72 ± 6.16 nM/mg protein) (*P* > 0.05) (Table 5), while a higher significant effect was shown by SL_22_ extract as it reduced the level of MDA down to 37.64 ± 12.80 nM/mg protein (compared to controls (*P*=0.005), to Met (*P*=0.0001), and to SL_22_ + Met (*P*=0.011) groups). Data suggest the significant role of the crude extracts in reducing levels of malondialdehyde and then the levels of lipid peroxidation in the treated mice brain.

## 4. Discussion

The present report assesses the effect of crude extracts from bacteria's culture media on neuropsychiatric behaviors, namely, memory impairment, anxiety, and depression-related behaviors, in mice. Cognitive deficits, negative symptoms, and some oxidative markers in methionine-induced mice models of schizophrenia were as well addressed. Two halophilic bacteria (strains SL_22_ and BM_20_) clustered within *Bacillus* and *Pseudomonas* genera, respectively, were selected as model bacteria. *Bacillus* and *Pseudomonas* genera, widely reported for their antioxidant activities, belong to two of the most reported phyla when studying the biodiversity of hypersaline environments, *Firmicutes* and *Proteobacteria*, respectively [58, 59]. Both genera constitute excellent sources of bioactive compounds that could cover a broad spectrum of industrial and medical applications. Moreover, considering their ability to withstand extreme conditions of salinity, pH, and temperature and to exhibit high activities at low concentrations, bacteria could, nowadays, challenge plants as producers of novel secondary metabolites of high interest.

In the present study, halophilic crude extracts were firstly assessed for their antioxidant activities. Both chosen bacteria (SL_22_ and BM_20_) were able to produce considerable amounts of phenol-containing and flavonoid-like compounds, which are well known for their biological activities, including modulation of neuropsychiatric disorders [60]. When compared to strain BM_20_, strain SL_22_ was able to produce higher quantities of phenol-containing compounds. However, strain BM_20_ showed higher production of flavonoid-like compounds, mostly responsible for the significant scavenging activity of the stable free radical DPPH and of the Mo(VI) reducing activities. The obtained results corroborate the fact that the antioxidant activity is a function of the polyphenol and flavonoid contents because of their activity to donate either protons or electrons to reduce free radicals [48, 61], and they are in line with previous studies that reported the potential antioxidant activity of extracts prepared from halophilic bacteria [38, 39, 62–64]. Because oxidative damage has been closely linked to neuropsychiatric disorders and a lot of reports have demonstrated the possible use of antioxidants in the treatment of such disorders, we got interested in testing the effect of those two extracts on short-term memory impairment, anxiety, and depression-like manifestations using three of the most reliable tests, Y-maze, elevated plus maze, and forced swimming test, respectively, as behavioral markers [65].

The assessment of short-term memory performance in the Y-maze task revealed that, generally, the administration of extracts at 120 mg/kg resulted in an increase of the spontaneous alternation percentage compared to 60 mg/kg administration (Figure 2(a)). Crude extract SL_22_ has consistently manifested a better positive effect regarding the improvement of spatial memory in comparison with extract BM_20_ that showed a very slight effect in terms of short-term memory enhancement. The increase in the number of arm entries following the oral administration of extracts at 60 mg/kg as compared with mice administered with doses of 120 mg/kg suggests a positive effect on exploratory and locomotor activities at low extract doses. The obtained results (Figure 2(b)) disclosed the effect of extracts on spatial working memory and locomotor activity in the central nervous system. The decrease in mice's exploratory and locomotor activities could be explained either by a potential myorelaxant effect of these extracts when administered at higher doses or by an impairment of the exploratory and/or locomotor behaviors in the corresponding treated mice. In this context, Foyet and his group [33] were interested in testing the effect of *Emilia coccinea* on the improvement of short-term memory in a dose-dependent manner. They noticed a significant augmentation of spatial memory in animals treated with a high dose of *Emilia coccinea* extract (400 mg/kg), which was reflected in an increased percentage of spontaneous alternations. However, the number of arm entries was decreased compared to that of mice receiving a less concentrated dose (200 mg/kg) of the same extract, suggesting sedative effect of the tested extract when administered at high doses. Even more, Foyet and his group compared the extract behavior to that of diazepam (a known anxiolytic and antidepressant medication that acts as a sedative at high doses) and found that mice exhibited the same behavior [33]. Elevated plus maze is used to evaluate anxiety-like behavior by considering time spent in open arms as a main index of anxiety as suggested by Ciobica et al. [52]. Our results indicate that SL_22_ and BM_20_ crude extracts manifested opposite effects. Mice treated with extract SL_22_ (120 mg/kg) spent more time in the open arms compared to those treated with extract SL_22_ (60 mg/kg), lasting longer than the control group, suggesting an anxiolytic effect of the crude extract SL_22_. Mice receiving BM_20_ extract at 60 mg/kg spent less time in the open arms than the controls and more time as compared to the BM_20_ (120 mg/kg) group, suggesting an anxiogenic effect of the crude extract BM_20_ (Figure 2(c)). In the FST, the depressive effect of BM_20_ extract administered at 120 mg/kg was remarkable when compared to the control group, whereas we noticed a potential antidepressant effect of BM_20_ (60 mg/kg) as compared to control animals (Figure 2(d)), which could be a false positive effect attributed to the stimulation of the locomotor activity by this extract [66], confirming the hyperactivity of mice administered with extract BM_20_ seen in the Y-maze test at the same administered dose. SL_22_ extract was found to significantly improve depression-like behavior by reducing immobility time for up to 7 seconds when administered at 60 mg/kg and 16 seconds when administered at 120 mg/kg, interestingly exceeding the effect of a reported classical antidepressant, namely, escitalopram, that reduces the duration of immobility for only 80 seconds [67]. Taken together, our results show that SL_22_ extract manifested positive effects on all the assessed behavioral tasks in a dose-dependent manner, which could be/is linked to a possible decrease in exploratory and/or locomotor activities at high doses. BM_20_ extract manifested, on the one hand, a slight effect on short-term memory enhancement coupled with a decrease in the exploratory and/or locomotor activities at 120 mg/kg administration, and on the other hand, possible anxiogenic and depressive effects in treated animals. Because of the high complexity of all assessed disorders, it is very difficult to suggest a precise mechanism of action of the tested bacterial extracts. In order to further understand the mechanisms of action of these bacterial crude extracts, there is a need for studying the effects of BM_20_ and SL_22_ crude extracts on the neuronal receptors involved in these disorders like serotonergic (5-HT1A) and dopaminergic receptors, measuring the oxidative stress markers in the temporal lobe after treatment, measuring the levels of monoamines (5HT, dopamine, and noradrenaline) in different areas of the brain, and/or elucidating their effects as probiotics which are widely reported for their effects on regulating the gut-microbiome imbalances identified as a main cause of the brain behavioral dysfunctions [36].

Moreover, both bacterial crude extracts were assessed (at 120 mg/kg) for their effects on negative symptoms (anxiety and depression), on cognitive deficits (loss of memory), and on oxidative stress state in methionine-induced mice models of schizophrenia, using elevated plus maze (anxiety), forced swimming test (depression), and Y-maze and NOR tests (short-term memory impairment). The assessment of spatial memory in the Y-maze confirms the short-term memory improvement effects of the tested crude extracts, implying their effects on the improvement of the cognitive deficits developed by methionine administration. Nonetheless, the decrease in the recorded number of arm entries suggests a negative effect of extracts on improving the locomotor activity of the corresponding mice, showing no effect on positive symptom improvement. Similar findings have been reported by Pitsikas in 2016 [68], who reported that acute administration of corcins (15–30 mg/kg), a constituent of saffron (*Crocus sativus*), reversed recognition memory deficits produced by the NMDA receptor antagonistic ketamine (KET) (3 mg/kg) in rats, suggesting an effect of the active constituent in schizophrenic-related cognition deficits [68]. In another recent study, morin (a flavonol) was investigated for its capability to reduce biomarkers of neuroinflammation and neurodegenerative in lipopolysaccharide (LPS)- and KET-induced schizophrenic-like behavior in mice models. The results showed that morin was able to improve memory deficit induced by LPS and KET administration via increasing the percentage of correct alternations in Y-maze as compared to the KET + LPS group [69]. Similar observations were made when we assessed the improvement of cognitive memory in the NOR test; the obtained results could suggest a positive effect of the crude extracts on improving cognitive symptoms of schizophrenia-like disorders in the generated mice models and align perfectly with a study carried out by El-Marasy and his group [70] who explored the memory-enhancing effects of *Nigella sativa* oil (NSO) and wheat germ oil (WGO) on scopolamine-induced amnesic rats. The study showed that NSO and WGO significantly reversed scopolamine-induced deficits of memory impairment as assessed in the NOR test in rats. NSO- and WGO-treated rats showed higher preference to explore the novel object than the familiar one, suggesting the effects of extracts on cognitive memory improvement [70]. Regarding the EPM test (Figure 4(a)), the extracts showed significant antianxiety effects at the time of the experiment; except for the SL_22_ group and compared to control and Met groups, all other groups spent more time in open arms. However, the fact that mice administered with SL_22_ extract were able to spend more time in the open arms than the Met group despite their less healthy state suggests or even confirms the anxiolytic effect of SL_22_ extract. Similarly, a recent study has demonstrated the anxiolytic effect of oxytocin in methionine-induced rat models of schizophrenia. The administration of oxytocin increased the time spent in open arms of the EPM in comparison with the Met group [19]. Moreover, the constituents of saffron were further investigated for their effects on negative symptoms of schizophrenia in rats and were found to attenuate the social isolation induced by a subchronic treatment with KET, which supports its effect on improving disorders related to negative symptoms of schizophrenia [71]. A reverse pattern was seen in the results for the FST test where mice belonging to the SL_22_ group were able to swim for almost all the test time, exhibiting therefore an effect on both positive and negative symptoms of schizophrenia (Figure 4(b)). However, as compared to controls, BM_20_ extract's administration resulted in increasing immobility time, suggesting a depressive effect of this crude extract. Similarly, *Galphimia glauca* extract and a galphimine-rich fraction were evaluated for their effects on KET-induced schizophrenic behavioral models in mice by means of the open field test, passive avoidance test, and forced swimming test. The tested extracts showed consistent interaction with the dopaminergic pathway and inhibited the deficit caused by KET in mice; since in FST, extracts were able to revert the effect of KET and reduce the immobility time of treated mice to an equal value to that of the baseline group without KET. Therefore, they suggested the ability of extracts to block both positive and cognitive symptoms associated with psychosis induced by KET [72]. While the complexity of the assessed disorders makes it difficult to suggest a precise mechanism of action of the tested bacterial crude extracts, the boosting effect of administration of Met to both SL_22_ and BM_20_ groups suggests a positive interaction between methionine and the bacterial crude extracts, which may be due to the interaction of compounds contained in the crude extracts in the methylation process conducted by methionine. Even though the behavioral approaches used in this study are highly reliable and approved by all the behavioral studies, they are not free of limitations. The major limitation being their high sensitivity to the environmental conditions and to the emotional and state of the animal model, which could significantly interfere with the measurements and their interpretation. Hence, further studies are needed to better understand first the precise mechanism of action of individual entities in both crude extracts with regard to the mechanisms of induction or reversion of schizophrenia-like symptoms.

Data from the antioxidant enzymes activities in animal's groups used in this study have been performed to assess the effect of the extract's administration on the oxidative stress status. In this regard, activities of key antioxidant enzymes such as superoxide dismutase (SOD), glutathione peroxidase (GPx), and catalase (CAT) boosted by the measure of end products of lipid peroxidation (MDA) [3, 73] have been determined. We mainly focused on two antioxidant enzymes (SOD and GPx) along with the levels of MDA in the cortical temporal lobe of generated models of schizophrenia. Results revealed significant increase of the activity of antioxidant enzymes (SOD and GPx) and the reduction of the levels of MDA in the brain samples of mice administered with bacterial crude extracts as compared to the methionine group which showed opposite results (Figure 5 and Table 5). Similar biochemical findings were reported in a study that aimed at evaluating the effect of the hydroalcoholic extract of *Emilia coccinea* (Sims) G. on improving memory deficits and oxidative stress damage in scopolamine-treated rats. The extract showed a significant improvement of memory impairment in Y-maze, in addition to a significant augmentation of the assessed antioxidant enzymes (SOD, GPx, and CAT), along with reduced levels of lipid peroxidation in the rat's whole brain homogenates. The study suggests a positive effect of the herbal extract on cognitive dysfunctions through the blockage of oxidative effect of scopolamine [74]. Other results on the ability of oxytocin to improve memory, anxiety, and oxidative stress biomarkers in a methionine-induced rat model of schizophrenia [19] showed an increase in the specific activity of GPx and in levels of MDA in the temporal lobe of rat models, but with no significant modifications in SOD specific activity, which was explained by the fact that SOD represents the first enzyme in contact with free radicals and its increase could be attributed to some compensatory actions [4]. Overall, the aforementioned reports reinforce the reliability of the biochemical measurements of oxidative stress as biomarkers of the manifested antischizophrenic-like disorders in generated animal models. The results of this study could indeed be an interesting starting point toward the determination of the active constituents of the assayed crude extracts as a first stage to elucidation of their mechanism of action in relation to schizophrenic-like disorders. Generally speaking, some studies have highlighted the potential use of plants and their bioactive components as potential treatments for schizophrenia-like behaviors [10, 75], and here we report for the use of a scalable and biodiverse source of compounds, with neuropsychiatric disorder and oxidative stress state modulation ability in schizophrenia-like disorder, represented by extracts from bacteria, mainly, halophilic bacteria.

## 5. Conclusions

The present study is the first of its kind to demonstrate the effect of crude extracts of two halophilic bacterial strains (*Pseudomonas zhaodongensis* strain *BM*_*20*_ and *Bacillus stratosphericus strain SL*_*22*_) on negative symptoms (anxiety and depression-like disorders), cognitive deficits (short-term memory impairment), and some oxidative parameters (SOD, GPx, and MDA) in methionine-induced mice models of schizophrenia. Regarding their antipsychotic-like disorder effects, both extracts seemed to significantly improve short-term memory, anxiety, and depression-induced disorders as shown in Y-maze and NOR, EPM, and FST, respectively, suggesting thus their effects on ameliorating both negative symptoms and cognitive dysfunctions in methionine-induced mice models of schizophrenia. Furthermore, the assessment of the biochemical measures of two antioxidant enzymes (SOD and GPx) and lipid peroxidation marker (MDA) levels, in the temporal cortical lobe, correlates perfectly with their manifested effects on amelioration of the behavioral disorders and proves their antioxidant therapy efficiency in the treatment of schizophrenic-like behaviors. Nonetheless, further investigations are needed in order to purify and to determine the active fraction from the extracts and its mechanism of action.

## Figures and Tables

**Table 1:** Design of the experiments of administration of bacterial crude extracts (SL_22_ and BM_20_) and number of mice considered for the behavioral experiments.

Groups	Number of mice
Control	6
SL_22_ (120 mg/kg)	6
SL_22_ (60 mg/kg)	6
BM_20_ (120 mg/kg)	6
BM_20_ (60 mg/kg)	6

**Table 2:** Design of the experiments of administration of bacterial crude extracts (SL_22_ and BM_20_) and number of mice considered to carry out the schizophrenia experiments.

Groups	Methionine	Control	BM_20_	BM_20_ + methionine	SL_22_	SL_22_ + methionine
Number of tested mice	6	5	5	5	5	5

**Table 3:** Morphological, physiological, and biochemical characteristics differentiating *Bacillus stratosphericus* strain SL_22_ and *Pseudomonas zhaodongensis* strain BM_20_ from other related species.

Characteristics	*B. stratosphericus SL*_*22*_	*B. licheniformis*	*B. sonorensis*	*B. aerius*	*B. aerophilus*	*P. zhaodongensis BM*_*20*_	*P. xanthomarina*	*P. kunmingensis*	*P. stutzeri*
Cell shape	Bacilli	Bacilli	Bacilli	Bacilli	Bacilli	Bacilli	Bacilli	Bacilli	Bacilli
Gram staining	+	+	+	+	+	−	−	−	−
Oxygen requirement	Aerobic	AAF	AAF	Aerobic	Aerobic	Aerobic	Aerobic	Aerobic	Aerobic
Catalase	+	+	+	+	+	+	+	+	+
NaCl tolerance (M)	2	1.2	0.3	2	2	1	1.3	1	1.2
Growth at									
10°C	+	−	−	+	+	+	+	+	+
40°C	+	+	+	−	−	+	−	+	+
50°C	+	+	+	−	−	−	−	−	−
H_2_S	−	nd	nd	−	nd	−	−	−	−
Gas	−	−	−	−	nd	−	nd	nd	nd
Motility	+	+	+	+	+	+	+	+	+
Sporulation	+	+	+	+	+	−	−	−	−
Hydrolysis of									
Carboxymethylcellulose	+	nd	nd	nd	nd	−	nd	nd	nd
Starch	−	+	+	+	nd	+	−	+	+
Pectin	−	+	nd	nd	nd	+	nd	nd	nd
DNA	+	nd	nd	nd	nd	−	−	nd	nd
Esculin	+	+	nd	+	nd	−	−	nd	nd
Gelatin	+	+	nd	+	+	−	−	−	−
Urea	+	d	nd	−	−	−	−	nd	nd
Growth on									
Saccharose	−	nd	nd	−	nd	+	nd	nd	nd
Maltose	−	nd	nd	−	nd	+	+	+	+
Raffinose	−	−	nd	−	+	+	nd	−	nd
Arabinose	+	−	nd	d	+	+	+	−	−
Fructose	+	nd	nd	nd	nd	−	+	−	+
Galactose	−	nd	nd	−	nd	±	−	−	−
D-Sorbitol	−	−	nd	+	+	+	nd	−	−
Ribose	±	nd	nd	nd	nd	±	nd	nd	−
Xylose	−	nd	nd	−	nd	−	nd	−	−
Glucose	+	nd	nd	+	nd	+	+	+	+
Lactose	+	nd	nd	−	nd	±	nd	−	−
Mannitol	+	nd	nd	nd	nd	+	+	+	d
Fermentation of									
Glucose	+	+	+	+	nd	+	−	nd	nd
Lactose	−	+	nd	+	nd	−	nd	nd	nd
Mannitol	+	+	+	+	nd	+	−	−	−

All tested sugars were added at 1% in M9 minimum medium. −, negative; ±, weakly positive; +, positive; nd, not determined; d, variable (*N* = 2).

**Table 4:** Determination of total phenolic compounds (TPC), total flavonoid compounds (TFC), DPPH scavenging, and molybdenum reduction activities of the halophilic bacterial crude extracts.

	SL_17′_	SL_22_	BM_20_	MM_2′_
TPC (*μ*g Eq gallic acid per mg of crude extract)	05.35 ± 0.01	30.80 ± 0.16	29.40 ± 0.22	12.00 ± 0.01
TFC (*μ*g Eq quercetin per mg of crude extract)	03.9 ± 0.01	04.30 ± 0.01	12.40 ± 0	02.50 ± 0
% of DPPH scavenging activity	81.60 ± 0.07	36.05 ± 0.46	81.84 ± 0.06	64.19 ± 0.37
Mo(VI) reduction activity (*μ*g Eq of ascorbic acid per mg of crude extract)	09.10 ± 0.07	17.90 ± 0.13	18.20 ± 0.06	07.80 ± 0.05
Range of tolerance to NaCl (M)	0–0.2	0–2	0-1	0-1

Gallic acid and quercetin were used as reference (*N* = 3 per extract) in TPC and TFC determination, respectively. Ascorbic acid was used as reference in DPPH radical scavenging assay and in Mo (VI) reduction assay (*N* = 2 per extract, respectively). Values are expressed as mean ± SD.

**Table 5:** Effects of methionine-induced mice models of schizophrenia and halophilic bacterial crude extract administration on superoxide dismutase (SOD) specific activity, glutathione peroxidase (GPx) specific activity, and malondialdehyde (MDA) concentration in the temporal lobe.

	Control	Methionine	BM_20_ + Met	BM_20_	SL_22_ + Met	SL_22_
Superoxide dismutase specific activity (U/mg protein)	1.23 ± 0.29	0.94 ± 0.38	1.20 ± 0.43	1.50 ± 0.30	1.41 ± 0.27	1.94 ± 0.21
Glutathione peroxidase specific activity (U/mg protein)	4.12 ± 0.37	3.12 ± 0.19	3.44 ± 0.29	4.25 ± 0.71	3.78 ± 0.41	4.88 ± 0.76
Malondialdehyde concentration (nM/mg protein)	69.74 ± 9.07	89.35 ± 8.76	71.64 ± 6.16	63.27 ± 6.11	66.00 ± 8.56	43.50 ± 12.80

Values are expressed as mean ± SD. *N* = 5 per group.

## Data Availability

The data used to support the findings of this study are included within the article.
